# 5-Methyl-1-[(4-methyl­phen­yl)sulfon­yl]-1*H*-pyrazol-3-yl 4-methyl­benzene­sulfonate

**DOI:** 10.1107/S1600536812027717

**Published:** 2012-06-23

**Authors:** Shahzad Murtaza, Naghmana Kausar, M. Nawaz Tahir, Javaria Tariq, Samaira Bibi

**Affiliations:** a University of Gujrat, Department of Chemistry, Hafiz Hayat Campus, Gujrat, Pakistan; b University of Sargodha, Department of Physics, Sargodha, Pakistan

## Abstract

In the title compound, C_18_H_18_N_2_O_5_S_2_, the tolyl rings are oriented at a dihedral angle of 16.15 (11)° with respect to one another. The 5-methyl-1*H*-pyrazol-3-ol ring is roughly planar (r.m.s. deviation = 0.0231 Å) and subtends angles of 73.82 (8) and 89.85 (8)° with the tolyl rings. In the crystal, very weak π–π inter­actions between tolyl groups, with centroid–centroid distances of 4.1364 (19) and 4.0630 (16) Å, together with a C—H⋯π contact generate a three-dimensional network.

## Related literature

For related structures, see: Gogoi *et al.* (2009 ▶); Murtaza *et al.* (2012 ▶).
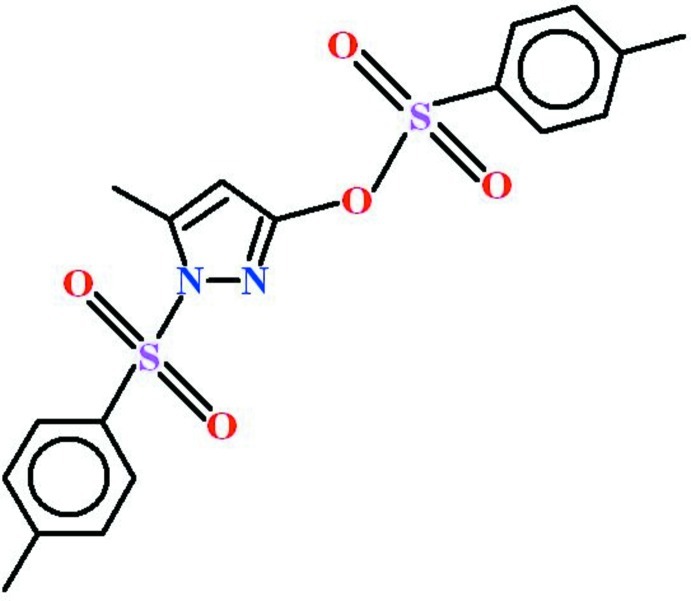


## Experimental

### 

#### Crystal data

C_18_H_18_N_2_O_5_S_2_
*M* *_r_* = 406.46
Monoclinic, 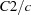

*a* = 22.296 (2) Å
*b* = 8.0444 (7) Å
*c* = 20.915 (2) Å
β = 98.521 (6)°
*V* = 3709.7 (6) Å^3^
*Z* = 8
Mo *K*α radiation
μ = 0.32 mm^−1^
*T* = 296 K
0.30 × 0.25 × 0.22 mm

#### Data collection

Bruker Kappa APEXII CCD diffractometer
Absorption correction: multi-scan (*SADABS*; Bruker, 2005 ▶) *T*_min_ = 0.910, *T*_max_ = 0.933
15506 measured reflections
4124 independent reflections
2548 reflections with *I* > 2σ(*I*)
*R*_int_ = 0.055

#### Refinement

*R*[*F* ^2^ > 2σ(*F* ^2^)] = 0.057
*wR*(*F* ^2^) = 0.128
*S* = 1.01
4124 reflections
247 parameters
H-atom parameters constrained
Δρ_max_ = 0.27 e Å^−3^
Δρ_min_ = −0.35 e Å^−3^

### 

Data collection: *APEX2* (Bruker, 2009 ▶); cell refinement: *SAINT* (Bruker, 2009 ▶); data reduction: *SAINT*; program(s) used to solve structure: *SHELXS97* (Sheldrick, 2008 ▶); program(s) used to refine structure: *SHELXL97* (Sheldrick, 2008 ▶); molecular graphics: *ORTEP-3 for Windows* (Farrugia, 1997 ▶) and *PLATON* (Spek, 2009 ▶); software used to prepare material for publication: *WinGX* (Farrugia, 1999 ▶) and *PLATON*.

## Supplementary Material

Crystal structure: contains datablock(s) global, I. DOI: 10.1107/S1600536812027717/sj5243sup1.cif

Structure factors: contains datablock(s) I. DOI: 10.1107/S1600536812027717/sj5243Isup2.hkl

Supplementary material file. DOI: 10.1107/S1600536812027717/sj5243Isup3.cml

Additional supplementary materials:  crystallographic information; 3D view; checkCIF report

## Figures and Tables

**Table 1:** Hydrogen-bond geometry (Å, °) *Cg*2 is the centroid of the C1–C6 benzene ring.

*D*—H⋯*A*	*D*—H	H⋯*A*	*D*⋯*A*	*D*—H⋯*A*
C18—H18b⋯*Cg*2^i^	0.96	2.66	3.471 (3)	142

Symmetry code: (i) 
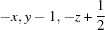
.

**Fig. 1.**
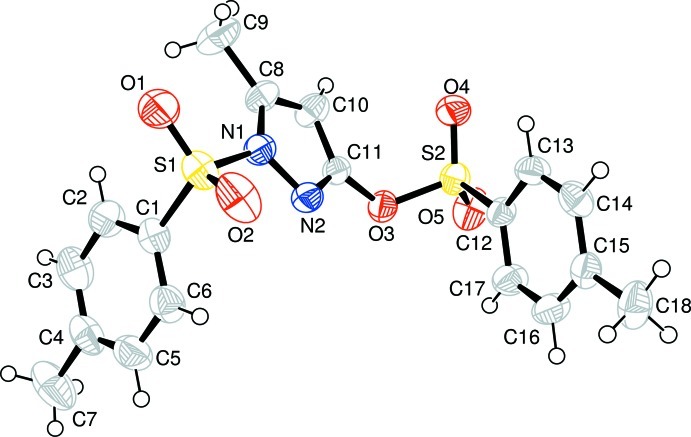
View of the title compound with the atom numbering scheme. The thermal ellipsoids are drawn at the 50% probability level. H-atoms are shown as small spheres of arbitrary radii.

## supplementary crystallographic information

### Comment

The title compound (I), (Fig. 1) has been synthesized as part of a study of enzyme inhibition and other biological activities of molecules incoorporating pyrazole moiety, an important component of many drugs. (I), was also prepared as a continuation of our work on sulfonyl derivatives, such as ethyl (3*E*)-3-[2-(4-bromophenylsulfonyl)hydrazin-1-ylidene]butanoate (Murtaza *et al.*, 2012). The crystal structure of 1(*R*)-4-(3-hydroxy-5-methyl-pyrazol-1-yl)-octan-2-one (Gogoi *et al.* 2009) has also been published and contains a 5-methyl-1*H*-pyrazol-3-ol unit similar to the one observed here.

In (I) , Fig. 1, the tolyl groups A (C1—C7) and B (C12—C18) are planar with r.m.s. deviations of 0.0137 and 0.0043 Å, respectively. The dihedral angle between the A/B planes is 16.15 (11)°. The central group, 5-methyl-1*H*-pyrazol-3-ol, C (C8—C11/N1/N2/O3) is also planar with an r.m.s. deviation of 0.0231 Å. The sulfonyl groups D (O1/S1/O2) and E (O4/S2/O5) are of course planar. The dihedral angles between A/C, A/D, A/E, B/C, B/D and B/E are 89.85 (8)°, 43.20 (9)°, 67.25 (13)°, 73.82 (8)°, 31.56 (9)° and 33.61 (12)°, respectively.

C18—H18b···π contacts form between dissimilar tolyl rings (Table 1). Weak π–π interactions are also found between like ring systems *Cg*2···*Cg*2^i^ [i = 1/2 - *x*, 1/2 - *y*, 1 - *z*] and *Cg*3···*Cg*3^ii^ [ii = - *x*, *y*, 1/2 - *z*] at distances of 4.1364 (19) and 4.0630 (16) %A respectively, where *Cg*2 and *Cg*3 are the centroids of the (C1—C6) and (C12—C17), benzene rings. These interactions play a role in stabilizing the structure, generating a three dimensional network.

### Experimental

A solution of 3-methyl-1*H*-pyrazol-5-ol (0.1 g, 1 mmol) was prepared in anhydrous tetrahydrofuran (THF) and NaH (0.048 g, 2 mmol) was added to it at room temperature. A separately prepared solution of 4-methyl benzenesulfonyl chloride (0.19 g, 0.001 mol) in THF (10 ml) was added dropwise to the above mixture. The mixture was stirred for 2 h and solvent was evaporated to yield white prisms of (I).

M. p. 483 K.

### Refinement

The H-atoms were positioned geometrically (C–H = 0.93–0.96 Å) and were included in the refinement in the riding model approximation, with *U*_iso_(H) = *xU*_eq_(C), where *x* = 1.5 for CH_3_ and *x* = 1.2 for other H-atoms.

### Crystal data

**Table:**

C_18_H_18_N_2_O_5_S_2_	*F*(000) = 1696
*M**_r_* = 406.46	*D*_x_ = 1.456 Mg m^−^^3^
Monoclinic, *C*2/*c*	Mo *K*α radiation, λ = 0.71073 Å
Hall symbol: -C 2yc	Cell parameters from 2548 reflections
*a* = 22.296 (2) Å	θ = 1.9–27.2°
*b* = 8.0444 (7) Å	µ = 0.32 mm^−^^1^
*c* = 20.915 (2) Å	*T* = 296 K
β = 98.521 (6)°	Prism, white
*V* = 3709.7 (6) Å^3^	0.30 × 0.25 × 0.22 mm
*Z* = 8	

### Data collection

**Table:**

Bruker Kappa APEXII CCD diffractometer	4124 independent reflections
Radiation source: fine-focus sealed tube	2548 reflections with *I* > 2σ(*I*)
Graphite monochromator	*R*_int_ = 0.055
Detector resolution: 7.80 pixels mm^-1^	θ_max_ = 27.2°, θ_min_ = 1.9°
ω scans	*h* = −28→26
Absorption correction: multi-scan (*SADABS*; Bruker, 2005)	*k* = −10→10
*T*_min_ = 0.910, *T*_max_ = 0.933	*l* = −26→26
15506 measured reflections	

### Refinement

**Table:**

Refinement on *F*^2^	Primary atom site location: structure-invariant direct methods
Least-squares matrix: full	Secondary atom site location: difference Fourier map
*R*[*F*^2^ > 2σ(*F*^2^)] = 0.057	Hydrogen site location: inferred from neighbouring sites
*wR*(*F*^2^) = 0.128	H-atom parameters constrained
*S* = 1.01	*w* = 1/[σ^2^(*F*_o_^2^) + (0.0531*P*)^2^ + 0.6607*P*] where *P* = (*F*_o_^2^ + 2*F*_c_^2^)/3
4124 reflections	(Δ/σ)_max_ = 0.001
247 parameters	Δρ_max_ = 0.27 e Å^−^^3^
0 restraints	Δρ_min_ = −0.35 e Å^−^^3^

### Special details

**Table:**

Geometry. Bond distances, angles *etc*. have been calculated using the rounded fractional coordinates. All su's are estimated from the variances of the (full) variance-covariance matrix. The cell e.s.d.'s are taken into account in the estimation of distances, angles and torsion angles
Refinement. Refinement of *F*^2^ against ALL reflections. The weighted *R*-factor *wR* and goodness of fit *S* are based on *F*^2^, conventional *R*-factors *R* are based on *F*, with *F* set to zero for negative *F*^2^. The threshold expression of *F*^2^ > σ(*F*^2^) is used only for calculating *R*-factors(gt) *etc*. and is not relevant to the choice of reflections for refinement. *R*-factors based on *F*^2^ are statistically about twice as large as those based on *F*, and *R*- factors based on ALL data will be even larger.

### Fractional atomic coordinates and isotropic or equivalent isotropic displacement parameters (Å^2^)

**Table:**

	*x*	*y*	*z*	*U*_iso_*/*U*_eq_	
S1	0.22735 (4)	0.04624 (9)	0.37485 (3)	0.0494 (3)	
S2	0.06192 (3)	0.04283 (8)	0.11235 (3)	0.0429 (2)	
O1	0.29151 (9)	0.0548 (3)	0.38078 (10)	0.0662 (8)	
O2	0.19810 (11)	−0.1021 (2)	0.38973 (10)	0.0691 (9)	
O3	0.08101 (8)	0.1414 (2)	0.17980 (9)	0.0460 (7)	
O4	0.11514 (9)	0.0014 (2)	0.08671 (10)	0.0550 (7)	
O5	0.01762 (9)	0.1490 (2)	0.07870 (10)	0.0614 (8)	
N1	0.20378 (10)	0.0816 (3)	0.29509 (10)	0.0418 (8)	
N2	0.14243 (10)	0.0735 (3)	0.27469 (11)	0.0421 (8)	
C1	0.19849 (13)	0.2141 (3)	0.41327 (12)	0.0435 (9)	
C2	0.22831 (14)	0.3644 (4)	0.41528 (14)	0.0540 (11)	
C3	0.20366 (17)	0.4966 (4)	0.44373 (16)	0.0641 (13)	
C4	0.15110 (16)	0.4839 (4)	0.46975 (14)	0.0571 (11)	
C5	0.12313 (15)	0.3307 (4)	0.46813 (14)	0.0610 (11)	
C6	0.14649 (14)	0.1940 (4)	0.44013 (13)	0.0529 (11)	
C7	0.12476 (17)	0.6330 (4)	0.49855 (16)	0.0890 (16)	
C8	0.23506 (12)	0.1375 (3)	0.24822 (14)	0.0439 (9)	
C9	0.30235 (13)	0.1595 (5)	0.25488 (17)	0.0719 (14)	
C10	0.19296 (12)	0.1638 (3)	0.19549 (14)	0.0476 (10)	
C11	0.13808 (12)	0.1233 (3)	0.21468 (13)	0.0365 (9)	
C12	0.02738 (11)	−0.1363 (3)	0.13625 (12)	0.0370 (8)	
C13	0.05269 (12)	−0.2897 (3)	0.12767 (13)	0.0452 (9)	
C14	0.02264 (14)	−0.4301 (3)	0.14397 (13)	0.0489 (10)	
C15	−0.03075 (13)	−0.4199 (3)	0.16949 (13)	0.0439 (9)	
C16	−0.05397 (13)	−0.2635 (4)	0.17760 (15)	0.0525 (11)	
C17	−0.02588 (12)	−0.1226 (3)	0.16106 (14)	0.0498 (10)	
C18	−0.06254 (15)	−0.5728 (4)	0.18785 (15)	0.0629 (11)	
H2	0.26411	0.37592	0.39787	0.0648*	
H3	0.22345	0.59864	0.44537	0.0767*	
H5	0.08773	0.31896	0.48629	0.0733*	
H6	0.12740	0.09105	0.43955	0.0635*	
H7A	0.15676	0.69601	0.52314	0.1338*	
H7B	0.09666	0.59725	0.52636	0.1338*	
H7C	0.10402	0.70111	0.46458	0.1338*	
H9A	0.31306	0.19893	0.21475	0.1079*	
H9B	0.32195	0.05485	0.26575	0.1079*	
H9C	0.31521	0.23876	0.28843	0.1079*	
H10	0.19958	0.20108	0.15503	0.0571*	
H13	0.08914	−0.29844	0.11129	0.0542*	
H14	0.03888	−0.53417	0.13754	0.0585*	
H16	−0.08991	−0.25389	0.19489	0.0630*	
H17	−0.04267	−0.01866	0.16655	0.0597*	
H18A	−0.04291	−0.66955	0.17383	0.0943*	
H18B	−0.10407	−0.57056	0.16754	0.0943*	
H18C	−0.06101	−0.57620	0.23394	0.0943*	

### Atomic displacement parameters (Å^2^)

**Table:**

	*U*^11^	*U*^22^	*U*^33^	*U*^12^	*U*^13^	*U*^23^
S1	0.0648 (6)	0.0397 (4)	0.0433 (4)	0.0083 (4)	0.0063 (4)	0.0018 (3)
S2	0.0445 (4)	0.0383 (4)	0.0456 (4)	−0.0034 (3)	0.0055 (3)	0.0012 (3)
O1	0.0546 (14)	0.0807 (16)	0.0598 (14)	0.0248 (12)	−0.0034 (11)	−0.0045 (12)
O2	0.1157 (19)	0.0378 (12)	0.0559 (13)	−0.0012 (12)	0.0201 (13)	0.0062 (10)
O3	0.0365 (11)	0.0416 (11)	0.0582 (12)	0.0028 (9)	0.0017 (9)	−0.0106 (9)
O4	0.0536 (13)	0.0568 (12)	0.0595 (13)	−0.0117 (10)	0.0242 (10)	−0.0075 (10)
O5	0.0614 (14)	0.0494 (12)	0.0672 (13)	0.0001 (10)	−0.0109 (11)	0.0170 (10)
N1	0.0385 (14)	0.0469 (14)	0.0401 (12)	−0.0011 (10)	0.0059 (11)	0.0008 (10)
N2	0.0385 (14)	0.0420 (13)	0.0472 (13)	−0.0038 (10)	0.0108 (11)	−0.0029 (10)
C1	0.0525 (19)	0.0386 (15)	0.0381 (14)	0.0019 (13)	0.0029 (13)	0.0020 (12)
C2	0.057 (2)	0.0473 (18)	0.0584 (18)	−0.0057 (15)	0.0110 (15)	0.0004 (15)
C3	0.088 (3)	0.0406 (18)	0.062 (2)	0.0000 (17)	0.006 (2)	−0.0058 (15)
C4	0.076 (2)	0.054 (2)	0.0385 (15)	0.0171 (18)	−0.0003 (16)	−0.0036 (14)
C5	0.065 (2)	0.077 (2)	0.0431 (16)	0.0067 (18)	0.0148 (15)	−0.0024 (16)
C6	0.064 (2)	0.0512 (19)	0.0443 (16)	−0.0074 (15)	0.0105 (15)	0.0002 (14)
C7	0.124 (3)	0.079 (3)	0.061 (2)	0.044 (2)	0.004 (2)	−0.015 (2)
C8	0.0373 (17)	0.0417 (16)	0.0534 (16)	−0.0076 (13)	0.0095 (14)	0.0074 (13)
C9	0.040 (2)	0.097 (3)	0.080 (2)	−0.0111 (18)	0.0128 (17)	0.018 (2)
C10	0.0427 (18)	0.0506 (18)	0.0497 (17)	−0.0090 (14)	0.0071 (14)	0.0124 (13)
C11	0.0338 (16)	0.0290 (14)	0.0473 (15)	−0.0018 (11)	0.0077 (12)	−0.0048 (12)
C12	0.0335 (15)	0.0333 (14)	0.0438 (14)	−0.0009 (12)	0.0042 (12)	0.0002 (11)
C13	0.0416 (17)	0.0442 (16)	0.0522 (16)	0.0055 (13)	0.0149 (13)	−0.0022 (13)
C14	0.063 (2)	0.0357 (16)	0.0491 (16)	0.0035 (14)	0.0120 (15)	−0.0033 (13)
C15	0.0488 (18)	0.0433 (16)	0.0387 (14)	−0.0092 (13)	0.0032 (13)	0.0009 (12)
C16	0.0391 (17)	0.0541 (19)	0.067 (2)	−0.0019 (15)	0.0167 (15)	0.0012 (15)
C17	0.0439 (18)	0.0376 (16)	0.070 (2)	0.0036 (13)	0.0156 (15)	−0.0027 (14)
C18	0.074 (2)	0.053 (2)	0.0589 (19)	−0.0227 (17)	0.0003 (17)	0.0030 (15)

### Geometric parameters (Å, º)

**Table:**

S1—O1	1.419 (2)	C13—C14	1.382 (4)
S1—O2	1.416 (2)	C14—C15	1.377 (4)
S1—N1	1.697 (2)	C15—C16	1.381 (4)
S1—C1	1.743 (3)	C15—C18	1.498 (4)
S2—O3	1.6193 (19)	C16—C17	1.364 (4)
S2—O4	1.413 (2)	C2—H2	0.9300
S2—O5	1.412 (2)	C3—H3	0.9300
S2—C12	1.742 (3)	C5—H5	0.9300
O3—C11	1.378 (3)	C6—H6	0.9300
N1—N2	1.373 (3)	C7—H7A	0.9600
N1—C8	1.361 (4)	C7—H7B	0.9600
N2—C11	1.307 (4)	C7—H7C	0.9600
C1—C2	1.378 (4)	C9—H9A	0.9600
C1—C6	1.371 (4)	C9—H9B	0.9600
C2—C3	1.373 (5)	C9—H9C	0.9600
C3—C4	1.367 (5)	C10—H10	0.9300
C4—C5	1.379 (5)	C13—H13	0.9300
C4—C7	1.500 (5)	C14—H14	0.9300
C5—C6	1.383 (4)	C16—H16	0.9300
C8—C9	1.497 (4)	C17—H17	0.9300
C8—C10	1.355 (4)	C18—H18A	0.9600
C10—C11	1.382 (4)	C18—H18B	0.9600
C12—C13	1.380 (3)	C18—H18C	0.9600
C12—C17	1.369 (4)		
			
O1—S1—O2	120.84 (14)	C14—C15—C18	121.3 (2)
O1—S1—N1	103.83 (12)	C16—C15—C18	121.1 (3)
O1—S1—C1	111.00 (14)	C15—C16—C17	122.1 (3)
O2—S1—N1	105.76 (12)	C12—C17—C16	119.1 (2)
O2—S1—C1	109.76 (13)	C1—C2—H2	121.00
N1—S1—C1	103.99 (12)	C3—C2—H2	121.00
O3—S2—O4	108.61 (11)	C2—C3—H3	119.00
O3—S2—O5	102.25 (11)	C4—C3—H3	119.00
O3—S2—C12	103.03 (11)	C4—C5—H5	119.00
O4—S2—O5	121.22 (12)	C6—C5—H5	119.00
O4—S2—C12	110.23 (11)	C1—C6—H6	121.00
O5—S2—C12	109.70 (12)	C5—C6—H6	121.00
S2—O3—C11	120.84 (16)	C4—C7—H7A	109.00
S1—N1—N2	116.53 (17)	C4—C7—H7B	109.00
S1—N1—C8	130.3 (2)	C4—C7—H7C	109.00
N2—N1—C8	112.7 (2)	H7A—C7—H7B	109.00
N1—N2—C11	102.3 (2)	H7A—C7—H7C	109.00
S1—C1—C2	119.0 (2)	H7B—C7—H7C	109.00
S1—C1—C6	119.4 (2)	C8—C9—H9A	109.00
C2—C1—C6	121.7 (3)	C8—C9—H9B	109.00
C1—C2—C3	118.1 (3)	C8—C9—H9C	109.00
C2—C3—C4	122.5 (3)	H9A—C9—H9B	109.00
C3—C4—C5	117.9 (3)	H9A—C9—H9C	109.00
C3—C4—C7	120.6 (3)	H9B—C9—H9C	109.00
C5—C4—C7	121.4 (3)	C8—C10—H10	127.00
C4—C5—C6	121.6 (3)	C11—C10—H10	127.00
C1—C6—C5	118.3 (3)	C12—C13—H13	121.00
N1—C8—C9	125.9 (3)	C14—C13—H13	121.00
N1—C8—C10	105.7 (2)	C13—C14—H14	119.00
C9—C8—C10	128.4 (3)	C15—C14—H14	119.00
C8—C10—C11	105.3 (2)	C15—C16—H16	119.00
O3—C11—N2	118.2 (2)	C17—C16—H16	119.00
O3—C11—C10	127.6 (2)	C12—C17—H17	120.00
N2—C11—C10	114.1 (2)	C16—C17—H17	120.00
S2—C12—C13	119.7 (2)	C15—C18—H18A	109.00
S2—C12—C17	119.17 (19)	C15—C18—H18B	109.00
C13—C12—C17	121.1 (2)	C15—C18—H18C	109.00
C12—C13—C14	118.5 (2)	H18A—C18—H18B	109.00
C13—C14—C15	121.7 (2)	H18A—C18—H18C	109.00
C14—C15—C16	117.6 (2)	H18B—C18—H18C	109.00
			
O1—S1—N1—N2	177.3 (2)	N2—N1—C8—C10	−1.1 (3)
O1—S1—N1—C8	−11.9 (3)	N1—N2—C11—O3	−175.7 (2)
O2—S1—N1—N2	49.2 (2)	N1—N2—C11—C10	−0.4 (3)
O2—S1—N1—C8	−140.0 (2)	S1—C1—C2—C3	177.7 (2)
C1—S1—N1—N2	−66.5 (2)	C6—C1—C2—C3	−1.6 (4)
C1—S1—N1—C8	104.4 (3)	S1—C1—C6—C5	−177.5 (2)
O1—S1—C1—C2	31.7 (3)	C2—C1—C6—C5	1.8 (4)
O1—S1—C1—C6	−148.9 (2)	C1—C2—C3—C4	0.0 (5)
O2—S1—C1—C2	167.9 (2)	C2—C3—C4—C5	1.4 (5)
O2—S1—C1—C6	−12.8 (3)	C2—C3—C4—C7	−178.1 (3)
N1—S1—C1—C2	−79.4 (2)	C3—C4—C5—C6	−1.2 (5)
N1—S1—C1—C6	100.0 (2)	C7—C4—C5—C6	178.3 (3)
O4—S2—O3—C11	−22.9 (2)	C4—C5—C6—C1	−0.4 (4)
O5—S2—O3—C11	−152.18 (18)	N1—C8—C10—C11	0.8 (3)
C12—S2—O3—C11	93.98 (19)	C9—C8—C10—C11	179.3 (3)
O3—S2—C12—C13	−116.7 (2)	C8—C10—C11—O3	174.6 (2)
O3—S2—C12—C17	65.8 (2)	C8—C10—C11—N2	−0.3 (3)
O4—S2—C12—C13	−0.9 (3)	S2—C12—C13—C14	−176.7 (2)
O4—S2—C12—C17	−178.5 (2)	C17—C12—C13—C14	0.8 (4)
O5—S2—C12—C13	135.0 (2)	S2—C12—C17—C16	177.7 (2)
O5—S2—C12—C17	−42.5 (3)	C13—C12—C17—C16	0.2 (4)
S2—O3—C11—N2	−123.4 (2)	C12—C13—C14—C15	−1.3 (4)
S2—O3—C11—C10	61.9 (3)	C13—C14—C15—C16	0.8 (4)
S1—N1—N2—C11	173.28 (18)	C13—C14—C15—C18	−179.2 (3)
C8—N1—N2—C11	0.9 (3)	C14—C15—C16—C17	0.2 (4)
S1—N1—C8—C9	9.3 (4)	C18—C15—C16—C17	−179.8 (3)
S1—N1—C8—C10	−172.1 (2)	C15—C16—C17—C12	−0.7 (5)
N2—N1—C8—C9	−179.6 (3)		

### Hydrogen-bond geometry (Å, º)

Cg2 is the centroid of the C1–C6 benzene ring.

**Table:**

*D*—H···*A*	*D*—H	H···*A*	*D*···*A*	*D*—H···*A*
C18—H18b···*Cg*2^i^	0.96	2.66	3.471 (3)	142

Symmetry code: (i) −*x*, *y*−1, −*z*+1/2.

## References

[bb1] Bruker (2005). *SADABS* Bruker AXS Inc., Madison, Wisconsin, USA.

[bb2] Bruker (2009). *APEX2* and *SAINT* Bruker AXS Inc., Madison, Wisconsin, USA.

[bb3] Farrugia, L. J. (1997). *J. Appl. Cryst.* **30**, 565.

[bb4] Farrugia, L. J. (1999). *J. Appl. Cryst.* **32**, 837–838.

[bb5] Gogoi, S., Zhao, C.-G. & Ding, D. (2009). *Org. Lett.* **11**, 2246–2252. 10.1021/ol900538q PMC2752151 19415906

[bb6] Murtaza, S., Kausar, N., Abbas, A., Tahir, M. N. & Zulfiqar, M. (2012). *Acta Cryst.* E**68**, o1616. 10.1107/S1600536812019265 PMC3379222 22719420

[bb7] Sheldrick, G. M. (2008). *Acta Cryst.* A**64**, 112–122. 10.1107/S0108767307043930 18156677

[bb8] Spek, A. L. (2009). *Acta Cryst.* D**65**, 148–155. 10.1107/S090744490804362X PMC2631630 19171970

